# Effect of blood alcohol level on outcome of patients with traumatic brain injury

**DOI:** 10.1186/cc13663

**Published:** 2014-03-17

**Authors:** V Borovni Lesjak, M Strnad, V Vujanovic Popovic, T Pelcl

**Affiliations:** 1 ZD dr. Adolfa Drolca Maribor, Slovenia

## Introduction

We investigated whether blood alcohol levels (BAL) had any impact on presentation and outcome in patients with traumatic brain injury (TBI).

## Methods

Forty-six patients with TBI requiring intubation between January 2000 and December 2012 were included. Patients were grouped into BAL-positive (>0.5%o; *n *= 24) and BAL-negative (<0.5%o; *n *= 22). Physiological parameters and outcome (survival to hospital discharge (STHD)) and neurological outcomes (Glasgow Outcome Scale (GOS), Cerebral Performance Category (CPC) and Glasgow Coma Scale (GCS)) were analyzed. Differences between groups were analyzed using Student's *t *test and results presented as mean ± SD.

## Results

There were no significant differences in gender and age distributions between the BAL-negative and BAL-positive groups (73% vs. 88% male; *P *= 0.218; 53 ± 21 vs. 43 ± 17 years; *P *= 0.098). There were also no differences in initial systolic blood pressure (BP; 144 ± 30 vs. 132 ± 37 mmHg; *P *= 0.241) and respiratory rate (RR; 13 ± 6 vs. 12 ± 7/ minute; *P *= 0.891) but BAL did affect the initial GCS score (7 ± 3 vs. 5 ± 2; *P *= 0.022). There was no effect on CPC (3.5 ± 1.9 vs. 2.8 ± 1.8; *P *= 0.185), GOS (2.4 ± 1.8 vs. 3.0 ± 1.7; *P *= 0.270), and GCS at discharge from the hospital (14 ± 2 vs. 14 ± 2; *P *= 0.801). There were also no differences in length of hospital stay (LOHS; 15 ± 23 vs. 23 ± 34; *P *= 0.372) and STHD (1.6 ± 0.5 vs. 1.3 ± 0.5; *P *= 0.083).

## Conclusion

BAL did not have a significant effect on presenting physiological parameters such as systolic BP and RR. It did, however, affect the presenting GCS score, which was significantly lower in the BAL-positive group. BAL did not seem to have an effect on functional outcome or mortality measured by STHD. No favorable neuroprotective or deleterious effect was observed, demonstrated by no significant differences in CPC, GOS or GCS scores at discharge.

